# 2-Chloro-1-(3-fluoro­benz­yloxy)-4-nitro­benzene

**DOI:** 10.1107/S160053680903431X

**Published:** 2009-09-05

**Authors:** Hui-ling Yu

**Affiliations:** a Yibin Vocational & Technical College, Si chuan, People’s Republic of China

## Abstract

In the title compound, C_13_H_9_ClFNO_3_, the benzene rings are oriented at a dihedral angle of 41.23 (5)°. In the crystal structure, inter­molecular C—H⋯O inter­actions link the mol­ecules in a herring-bone arrangement along the *b* axis and weak π–π contacts between the benzene rings [centroid–centroid distance = 3.881 (1) Å] may further stabilize the structure.

## Related literature

The title compound is a dual ErbB-1/ErbB-2 tyrosine kinase inhibitor, see: Petrov *et al.* (2006 ▶). For bond-length data, see: Allen *et al.* (1987 ▶).
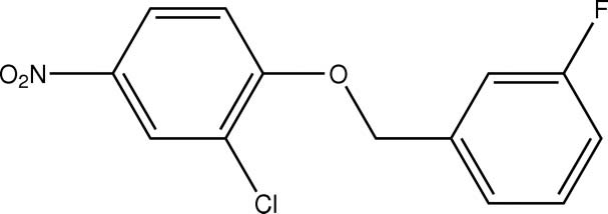


## Experimental

### 

#### Crystal data

C_13_H_9_ClFNO_3_
*M* *_r_* = 281.66
Monoclinic, 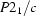

*a* = 8.3290 (17) Å
*b* = 12.640 (3) Å
*c* = 11.875 (2) Å
β = 96.94 (3)°
*V* = 1241.0 (4) Å^3^
*Z* = 4
Mo *K*α radiation
μ = 0.32 mm^−1^
*T* = 294 K
0.30 × 0.20 × 0.10 mm

#### Data collection

Enraf–Nonius CAD-4 diffractometer
Absorption correction: ψ scan (North *et al.*, 1968 ▶) *T*_min_ = 0.909, *T*_max_ = 0.968
2411 measured reflections
2248 independent reflections
1340 reflections with *I* > 2σ(*I*)
*R*_int_ = 0.028
3 standard reflections frequency: 120 min intensity decay: 1%

#### Refinement

*R*[*F* ^2^ > 2σ(*F* ^2^)] = 0.052
*wR*(*F* ^2^) = 0.149
*S* = 1.01
2248 reflections
172 parameters
H-atom parameters constrained
Δρ_max_ = 0.16 e Å^−3^
Δρ_min_ = −0.25 e Å^−3^

### 

Data collection: *CAD-4 Software* (Enraf–Nonius, 1989 ▶); cell refinement: *CAD-4 Software*; data reduction: *XCAD4* (Harms & Wocadlo, 1995 ▶); program(s) used to solve structure: *SHELXS97* (Sheldrick, 2008 ▶); program(s) used to refine structure: *SHELXL97* (Sheldrick, 2008 ▶); molecular graphics: *ORTEP-3 for Windows* (Farrugia, 1997 ▶) and *PLATON* (Spek, 2009 ▶); software used to prepare material for publication: *SHELXL97* and *PLATON*.

## Supplementary Material

Crystal structure: contains datablocks global, I. DOI: 10.1107/S160053680903431X/hk2758sup1.cif

Structure factors: contains datablocks I. DOI: 10.1107/S160053680903431X/hk2758Isup2.hkl

Additional supplementary materials:  crystallographic information; 3D view; checkCIF report

## Figures and Tables

**Table 1:** Hydrogen-bond geometry (Å, °)

*D*—H⋯*A*	*D*—H	H⋯*A*	*D*⋯*A*	*D*—H⋯*A*
C7—H7*A*⋯O2^i^	0.97	2.49	3.423 (4)	162

Symmetry code: (i) 
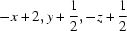
.

**Fig. 1.**
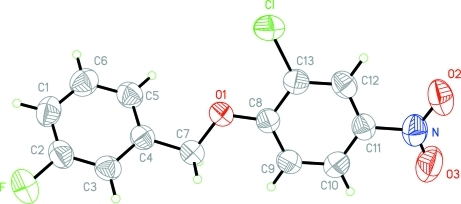
The molecular structure of the title molecule with the atom-numbering scheme. Displacement ellipsoids are drawn at the 50% probability level.

**Fig. 2.**
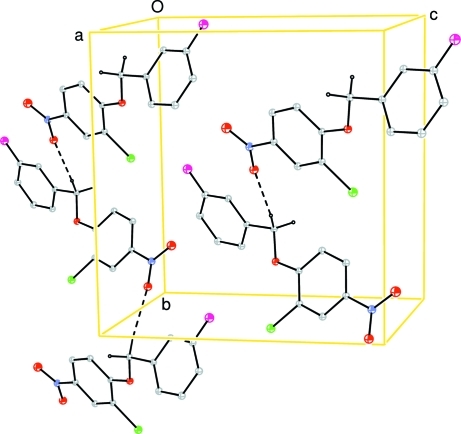
A partial packing diagram. Hydrogen bonds are shown as dashed lines.

## supplementary crystallographic information

### Comment

The title compound is one kind of important pharmaceutical intermediates, which is dual ErbB-1/ErbB-2 tyrosine kinase inhibitior (Petrov *et al.*, 2006). We report herein its crystal structure.

In the molecule of the title compound, (Fig. 1), the bond lengths (Allen *et al.*,  1987) and angles are within normal ranges. Rings A (C1-C6) and B (C8-C13) are, of course, planar and they are oriented at a dihedral angle of A/B = 41.23 (5)°. Atom C7 is -0.061 (3) Å away from the plane of ring A, while atoms Cl, O1, N and C7 are -0.007 (3), 0.001 (3), 0.018 (3) and 0.029 (3) Å away from the plane of ring B, respectively.

In the crystal structure, intermolecular C-H···O interactions link the molecules in herring-bone arrangement along the b axis and π–π contact between the benzene rings, Cg1—Cg2^i^, [symmetry code: (i) x, 1/2 - y, 1/2 + z, where Cg1 and Cg2 are centroids of the rings A (C1-C6) and B (C8-C13), respectively] may further stabilize the structure, with centroid-centroid distance of 3.881 (1) Å.

### Experimental

For the preparation of the title compound, in the presence of sodium carbonate (10 g), 2-chloro-4-nitrophenol (1 mmol) and 1-(bromomethyl)-3-fluorobenzene (1 mmol) in acetonitrile (25 ml) were stirred at 313 K for 8 h. Sodium carbonate was filtered off and the filtrate was washed with brine. The organic phase was dried over anhydrous sodium sulfate, filtered and concentrated to give the crude product, which was crystallized from ethyl acetate to give the title compound. Crystals suitable for X-ray analysis were obtained by dissolving the title compound (0.1 g) in ethyl acetate (10 ml) and evaporating the solvent slowly at room temperature for 3 d.

### Refinement

H atoms were positioned geometrically with C-H = 0.93 and 0.97 Å for aromatic and methylene H atoms, respectively, and constrained to ride on their parent atoms, with U_iso_(H) = 1.2U_eq_(C).

### Crystal data

**Table:**

C_13_H_9_ClFNO_3_	*F*(000) = 576
*M**_r_* = 281.66	*D*_x_ = 1.508 Mg m^−^^3^
Monoclinic, *P*2_1_/*c*	Mo *K*α radiation, λ = 0.71073 Å
Hall symbol: -P 2ybc	Cell parameters from 25 reflections
*a* = 8.3290 (17) Å	θ = 9–12°
*b* = 12.640 (3) Å	µ = 0.32 mm^−^^1^
*c* = 11.875 (2) Å	*T* = 294 K
β = 96.94 (3)°	Block, yellow
*V* = 1241.0 (4) Å^3^	0.30 × 0.20 × 0.10 mm
*Z* = 4	

### Data collection

**Table:**

Enraf–Nonius CAD-4 diffractometer	1340 reflections with *I* > 2σ(*I*)
Radiation source: fine-focus sealed tube	*R*_int_ = 0.028
graphite	θ_max_ = 25.3°, θ_min_ = 2.4°
ω/2θ scans	*h* = 0→10
Absorption correction: ψ scan (North *et al.*, 1968)	*k* = 0→15
*T*_min_ = 0.909, *T*_max_ = 0.968	*l* = −14→14
2411 measured reflections	3 standard reflections every 120 min
2248 independent reflections	intensity decay: 1%

### Refinement

**Table:**

Refinement on *F*^2^	Primary atom site location: structure-invariant direct methods
Least-squares matrix: full	Secondary atom site location: difference Fourier map
*R*[*F*^2^ > 2σ(*F*^2^)] = 0.052	Hydrogen site location: inferred from neighbouring sites
*wR*(*F*^2^) = 0.149	H-atom parameters constrained
*S* = 1.01	*w* = 1/[σ^2^(*F*_o_^2^) + (0.07*P*)^2^] where *P* = (*F*_o_^2^ + 2*F*_c_^2^)/3
2248 reflections	(Δ/σ)_max_ < 0.001
172 parameters	Δρ_max_ = 0.16 e Å^−^^3^
0 restraints	Δρ_min_ = −0.25 e Å^−^^3^

### Special details

**Table:**

Geometry. All esds (except the esd in the dihedral angle between two l.s. planes) are estimated using the full covariance matrix. The cell esds are taken into account individually in the estimation of esds in distances, angles and torsion angles; correlations between esds in cell parameters are only used when they are defined by crystal symmetry. An approximate (isotropic) treatment of cell esds is used for estimating esds involving l.s. planes.
Refinement. Refinement of F^2^ against ALL reflections. The weighted R-factor wR and goodness of fit S are based on F^2^, conventional R-factors R are based on F, with F set to zero for negative F^2^. The threshold expression of F^2^ > 2sigma(F^2^) is used only for calculating R-factors(gt) etc. and is not relevant to the choice of reflections for refinement. R-factors based on F^2^ are statistically about twice as large as those based on F, and R- factors based on ALL data will be even larger.

### Fractional atomic coordinates and isotropic or equivalent isotropic displacement parameters (Å^2^)

**Table:**

	*x*	*y*	*z*	*U*_iso_*/*U*_eq_	
Cl	0.81771 (13)	0.51262 (7)	0.07511 (8)	0.0802 (4)	
F	0.4335 (3)	1.00189 (17)	−0.29984 (19)	0.0996 (8)	
O1	0.6878 (3)	0.72025 (15)	0.07000 (17)	0.0582 (6)	
O2	1.1588 (3)	0.5447 (2)	0.4720 (2)	0.0846 (8)	
O3	1.0836 (4)	0.6867 (3)	0.5501 (2)	0.1058 (10)	
N	1.0804 (3)	0.6268 (3)	0.4700 (3)	0.0694 (8)	
C1	0.3405 (4)	0.8293 (3)	−0.2781 (3)	0.0670 (10)	
H1A	0.2801	0.8325	−0.3492	0.080*	
C2	0.4320 (4)	0.9129 (3)	−0.2358 (3)	0.0629 (9)	
C3	0.5233 (4)	0.9117 (2)	−0.1320 (3)	0.0550 (8)	
H3A	0.5858	0.9700	−0.1069	0.066*	
C4	0.5210 (4)	0.8224 (2)	−0.0653 (3)	0.0513 (8)	
C5	0.4296 (4)	0.7364 (3)	−0.1061 (3)	0.0630 (9)	
H5A	0.4279	0.6756	−0.0621	0.076*	
C6	0.3407 (4)	0.7401 (3)	−0.2118 (3)	0.0707 (10)	
H6A	0.2801	0.6815	−0.2386	0.085*	
C7	0.6137 (4)	0.8219 (2)	0.0507 (3)	0.0587 (9)	
H7A	0.6959	0.8767	0.0564	0.070*	
H7B	0.5415	0.8358	0.1073	0.070*	
C8	0.7811 (4)	0.7033 (2)	0.1694 (3)	0.0492 (8)	
C9	0.8086 (4)	0.7766 (2)	0.2567 (3)	0.0564 (8)	
H9A	0.7609	0.8431	0.2488	0.068*	
C10	0.9064 (4)	0.7510 (3)	0.3551 (3)	0.0597 (9)	
H10A	0.9250	0.8002	0.4135	0.072*	
C11	0.9755 (4)	0.6531 (2)	0.3662 (3)	0.0531 (8)	
C12	0.9495 (4)	0.5787 (3)	0.2808 (3)	0.0575 (8)	
H12A	0.9970	0.5122	0.2896	0.069*	
C13	0.8532 (4)	0.6039 (2)	0.1836 (3)	0.0530 (8)	

### Atomic displacement parameters (Å^2^)

**Table:**

	*U*^11^	*U*^22^	*U*^33^	*U*^12^	*U*^13^	*U*^23^
Cl	0.1074 (8)	0.0574 (6)	0.0736 (7)	0.0078 (5)	0.0026 (5)	−0.0116 (4)
F	0.135 (2)	0.0791 (15)	0.0789 (15)	0.0098 (14)	−0.0083 (14)	0.0259 (11)
O1	0.0683 (14)	0.0475 (12)	0.0568 (14)	0.0075 (11)	−0.0005 (11)	0.0012 (10)
O2	0.0635 (17)	0.094 (2)	0.0931 (19)	0.0152 (16)	−0.0032 (14)	0.0228 (16)
O3	0.109 (2)	0.124 (3)	0.0751 (19)	0.014 (2)	−0.0258 (17)	−0.0135 (19)
N	0.0542 (18)	0.083 (2)	0.069 (2)	−0.0014 (18)	0.0016 (16)	0.0110 (18)
C1	0.061 (2)	0.083 (3)	0.055 (2)	0.011 (2)	−0.0022 (17)	−0.0035 (19)
C2	0.067 (2)	0.060 (2)	0.062 (2)	0.0124 (19)	0.0055 (18)	0.0090 (18)
C3	0.0521 (19)	0.0494 (18)	0.063 (2)	0.0055 (15)	0.0063 (16)	−0.0019 (15)
C4	0.0482 (18)	0.0511 (18)	0.0552 (19)	0.0061 (15)	0.0087 (15)	0.0009 (15)
C5	0.067 (2)	0.057 (2)	0.065 (2)	−0.0028 (18)	0.0075 (18)	0.0064 (17)
C6	0.061 (2)	0.075 (2)	0.074 (3)	−0.0052 (19)	0.001 (2)	−0.008 (2)
C7	0.068 (2)	0.0494 (19)	0.057 (2)	0.0020 (16)	0.0005 (17)	0.0031 (15)
C8	0.0461 (18)	0.0499 (18)	0.0515 (18)	−0.0016 (15)	0.0054 (15)	0.0030 (15)
C9	0.062 (2)	0.0480 (17)	0.060 (2)	0.0025 (16)	0.0068 (17)	0.0020 (16)
C10	0.060 (2)	0.063 (2)	0.056 (2)	−0.0072 (17)	0.0062 (17)	−0.0032 (16)
C11	0.0446 (18)	0.0566 (19)	0.058 (2)	−0.0012 (16)	0.0055 (15)	0.0078 (16)
C12	0.049 (2)	0.057 (2)	0.068 (2)	0.0068 (16)	0.0107 (17)	0.0077 (17)
C13	0.0534 (19)	0.0518 (19)	0.0548 (19)	−0.0026 (16)	0.0096 (16)	0.0017 (15)

### Geometric parameters (Å, °)

**Table:**

Cl—C13	1.728 (3)	C5—C6	1.379 (5)
F—C2	1.359 (4)	C5—H5A	0.9300
O1—C7	1.432 (3)	C6—H6A	0.9300
O1—C8	1.350 (4)	C7—H7A	0.9700
N—O2	1.225 (4)	C7—H7B	0.9700
N—O3	1.214 (4)	C8—C9	1.388 (4)
N—C11	1.460 (4)	C8—C13	1.394 (4)
C1—C2	1.363 (5)	C9—C10	1.380 (5)
C1—C6	1.375 (4)	C9—H9A	0.9300
C1—H1A	0.9300	C10—C11	1.366 (4)
C2—C3	1.367 (4)	C10—H10A	0.9300
C3—C4	1.381 (4)	C11—C12	1.380 (4)
C3—H3A	0.9300	C12—C13	1.361 (4)
C4—C5	1.382 (4)	C12—H12A	0.9300
C4—C7	1.496 (4)		
			
C8—O1—C7	118.3 (2)	O1—C7—H7A	110.0
O2—N—C11	118.2 (3)	C4—C7—H7A	110.0
O3—N—O2	123.5 (3)	O1—C7—H7B	110.0
O3—N—C11	118.3 (3)	C4—C7—H7B	110.0
C2—C1—C6	117.6 (3)	H7A—C7—H7B	108.4
C2—C1—H1A	121.2	O1—C8—C9	124.9 (3)
C6—C1—H1A	121.2	O1—C8—C13	116.3 (3)
F—C2—C1	118.5 (3)	C9—C8—C13	118.8 (3)
F—C2—C3	118.1 (3)	C10—C9—C8	120.3 (3)
C1—C2—C3	123.3 (3)	C10—C9—H9A	119.9
C2—C3—C4	118.7 (3)	C8—C9—H9A	119.9
C2—C3—H3A	120.6	C11—C10—C9	119.5 (3)
C4—C3—H3A	120.6	C11—C10—H10A	120.3
C3—C4—C5	119.2 (3)	C9—C10—H10A	120.3
C3—C4—C7	119.4 (3)	C10—C11—C12	121.3 (3)
C5—C4—C7	121.4 (3)	C10—C11—N	119.3 (3)
C6—C5—C4	120.3 (3)	C12—C11—N	119.4 (3)
C6—C5—H5A	119.8	C13—C12—C11	119.2 (3)
C4—C5—H5A	119.8	C13—C12—H12A	120.4
C1—C6—C5	120.8 (3)	C11—C12—H12A	120.4
C1—C6—H6A	119.6	C12—C13—C8	120.9 (3)
C5—C6—H6A	119.6	C12—C13—Cl	120.4 (3)
O1—C7—C4	108.4 (2)	C8—C13—Cl	118.6 (2)
			
C6—C1—C2—F	−180.0 (3)	C13—C8—C9—C10	0.3 (5)
C6—C1—C2—C3	0.3 (5)	C8—C9—C10—C11	−0.2 (5)
F—C2—C3—C4	179.1 (3)	C9—C10—C11—C12	−0.1 (5)
C1—C2—C3—C4	−1.2 (5)	C9—C10—C11—N	179.3 (3)
C2—C3—C4—C5	1.3 (5)	O3—N—C11—C10	11.0 (5)
C2—C3—C4—C7	−177.0 (3)	O2—N—C11—C10	−170.0 (3)
C3—C4—C5—C6	−0.5 (5)	O3—N—C11—C12	−169.6 (3)
C7—C4—C5—C6	177.7 (3)	O2—N—C11—C12	9.5 (4)
C2—C1—C6—C5	0.5 (5)	C10—C11—C12—C13	0.2 (5)
C4—C5—C6—C1	−0.4 (5)	N—C11—C12—C13	−179.2 (3)
C8—O1—C7—C4	178.5 (2)	C11—C12—C13—C8	−0.1 (4)
C3—C4—C7—O1	−140.2 (3)	C11—C12—C13—Cl	−179.9 (2)
C5—C4—C7—O1	41.6 (4)	O1—C8—C13—C12	−179.9 (3)
C7—O1—C8—C9	1.5 (4)	C9—C8—C13—C12	−0.2 (4)
C7—O1—C8—C13	−178.8 (3)	O1—C8—C13—Cl	−0.1 (4)
O1—C8—C9—C10	−180.0 (3)	C9—C8—C13—Cl	179.6 (2)

### Hydrogen-bond geometry (Å, °)

**Table:**

*D*—H···*A*	*D*—H	H···*A*	*D*···*A*	*D*—H···*A*
C7—H7A···O2^i^	0.97	2.49	3.423 (4)	162

Symmetry codes: (i) −*x*+2, *y*+1/2, −*z*+1/2.
